# Expression and Function of ZEB1 in the Cornea

**DOI:** 10.3390/cells10040925

**Published:** 2021-04-16

**Authors:** Yingnan Zhang, Xiao Liu, Wei Liang, Douglas C. Dean, Lijun Zhang, Yongqing Liu

**Affiliations:** 1 Department of Medicine, University of Louisville School of Medicine, Louisville, KY 40202, USA; yingnan.zhang@louisville.edu (Y.Z.); dcdean01@louisville.edu (D.C.D.); 2 James Brown Cancer Center, University of Louisville School of Medicine, Louisville, KY 40202, USA; 3 Department of Ophthalmology, The Second Xiangya Hospital of Central South China University, Changsha 410011, China; liuxiao-doctor@163.com; 4 Department of Ophthalmology, The Third People’s Hospital of Dalian, Dalian Medical University, Dalian 116033, China; liangwei_6227327@126.com; 5 Birth Defects Center, University of Louisville School of Dentistry, Louisville, KY 40202, USA

**Keywords:** ZEB1, corneal dystrophies, stem cell homeostasis, wound healing, inflammation, neovascularization

## Abstract

ZEB1 is an important transcription factor for epithelial to mesenchymal transition (EMT) and in the regulation of cell differentiation and transformation. In the cornea, ZEB1 presents in all three layers: the epithelium, the stroma and the endothelium. Mutations of ZEB1 have been linked to multiple corneal genetic defects, particularly to the corneal dystrophies including keratoconus (KD), Fuchs endothelial corneal dystrophy (FECD), and posterior polymorphous corneal dystrophy (PPCD). Accumulating evidence indicates that dysfunction of ZEB1 may affect corneal stem cell homeostasis, and cause corneal cell apoptosis, stromal fibrosis, angiogenesis, squamous metaplasia. Understanding how ZEB1 regulates the initiation and progression of these disorders will help us in targeting ZEB1 for potential avenues to generate therapeutics to treat various ZEB1-related disorders.

## 1. ZEB1 and Epithelial to Mesenchymal Transition (EMT)

The zinc-finger E homeobox-binding (ZEB) protein family of transcription factors (TFs) are composed of two members: ZEB1 and ZEB2, and best known for their role in driving epithelial to mesenchymal transition (EMT) by inhibiting the expression of the epithelial anchor protein E-cadherin (CDH1), a prominent tight junction protein for connecting epithelial cells. In recent years, our understanding of these transcription factors has been expanded, and it is clear that they are expressed in a variety of neural cells, immune cells, mesenchymal cells and endothelial cells. In these cells, ZEBs play important roles in regulation of cell differentiation, stemness, survival, and proliferation [1].

In 1993, when ZEB1, also termed TCF8 and δEF1, was first identified specifically expressing in the nervous system and the lens of the mesoderm tissue of chicken embryos, as a repressor of δ1-crystallin inhibitor and was considered to be involved in embryogenesis [2]. Later, a mouse model with Zeb1 deletion revealed the role of ZEB1 in development as ZEB1 is a master regulator of EMT in controlling the activation of EMT [3,4]. Its dysregulation has been observed in various cancer types and other pathological processes [5]. ZEB1 is characterized by an amino-terminal (NZF) and a carboxy-terminal (CZF) zinc finger cluster, and a homeodomain (HD) in the center. Other protein binding domains include SMAD binding domain (SBD), p300-P/CAF binding domain (CBD) and CtBP interacting domain (CID) [6]. The predominant mechanism by which ZEB1 represses gene expression is through an active transcriptional repression. ZEB1 can inhibit gene transcription by directly targeting the 5′-CANNTG-3′ E-box sequence located in gene promoters. It is worth noting that the two repressor domains of ZEB1 target different transcription factors and regulate the differentiation of specific tissues [7,8]. In addition, ZEB1 expression is regulated by multiple signaling pathways and components, including TGFβ, WNT, miRNAs and other factors [9].

EMT was initially observed during embryogenesis and plays an important role in early development, including neural and heart valve development, mesoderm and secondary palate formation [10,11,12]. It is recognized that the EMT and its reverse process can be resurrected in adult tissues under a variety of disease conditions including wound healing, fibrosis and cancer [13]. In many epithelial-derived malignancies, after receiving microenvironmental stimuli such as WNT, tumor necrosis factor-α (TNFα) and transforming growth factor beta (TGFβ), epithelial cells lose their ability to adhere to each other, acquire mesenchymal cell characteristics, and become more mobilizable [14]. In addition, EMT is also a key feature of pathological fibrotic responses to injuries, e.g., wound healing and fibrotic organ diseases [15]. During embryogenesis and carcinogenesis, epithelial cells convert from their differentiated state to undifferentiated mesenchymal cell-like state. But, knockdown of ZEB1 can completely reverse this EMT [16].

The growing literature shows that ZEB1 also plays a role in regulation of many physiological and pathological processes of the cornea. In the next section, we will summarize the roles of ZEB1 in regulation of corneal epithelial, stromal and endothelial diseases, and related pathogenic conditions such as corneal wound healing, inflammation and neovascularization.

## 2. The Cornea and ZEB1

**Figure 1:**
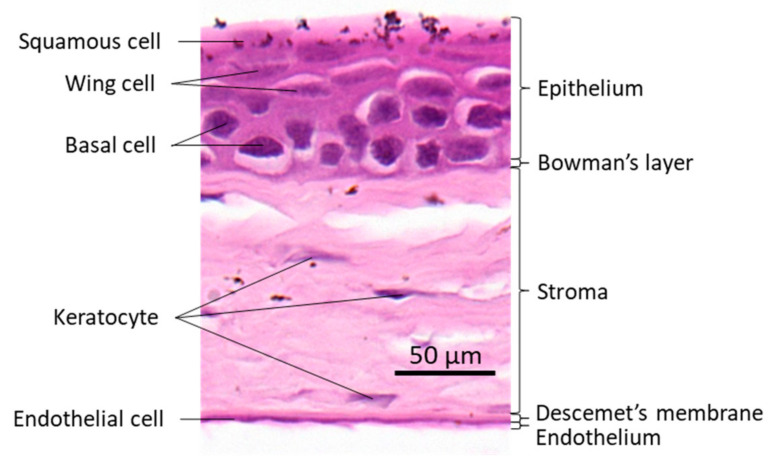
Mouse corneal structure and cell types.

**Figure 2:**
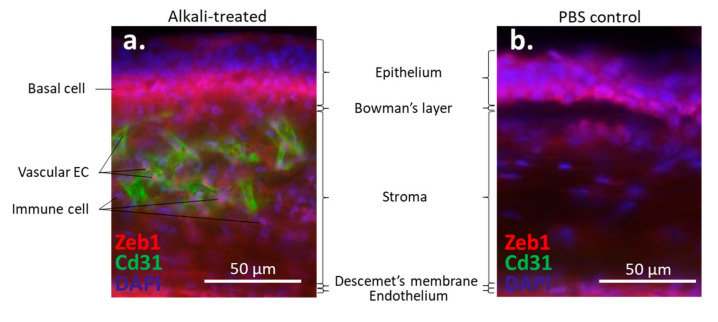
Alkali-induced mouse corneal neovascularization (NV). (**a**) In the alkali-treated cornea, Zeb1 is highly expressed in epithelial basal cells (BC), Cd31-expressing vascular endothelial cells (EC), and infiltrated immune cells (IC). (**b**) In the PBS control cornea, no Cd31-positive EC and IC are detected.

The cornea is a transparent avascular tissue that acts as a barrier and protects structures inside the eye. The mammalian cornea is composed of five layers including the epithelium, Bowman’s membrane, the stroma, Descemet’s membrane, and the endothelium (Figure 1). The epithelium is a five-to-six cell layer structure with three epithelial cell types: superficial, wing, and basal cells. The superficial cells are two to three layers of flat polygonal cells. The microvilli on the superficial surface increase corneal surface area that would more efficiently allow oxygen and other nutrients from tears diffusing into the cornea. The wing cells are two to three layers of wing-like shape cells and derived from the basal cells. The basal cells are single layer of cuboidal or columnar cells that have abundant organelles and are active mitotically, also known as transient amplifying (TA) cells. The corneal epithelium is the outmost tissue of the eye that is often bombarded by environmental physical, chemical, and pathogenic insults, sheared and restored constantly. Its cellular homeostasis is maintained by a group of stem cells located at the limbus, a narrow area between the cornea and sclera. These so-called limbal epithelial stem cells (LESC) not only renew themselves locally at the limbus, but also generate TA cells that can move along the Bowman’s membrane towards the corneal center and at the same time move up to the superficial layer to differentiate into epithelial cells simultaneously [17]. The corneal stromal fibroblasts, known as keratocytes, can also be regenerated though at a relatively slower pace [18]. The keratocyte regeneration process however, is not as clear and its reproduction is supposed to be either locally and/or come from the progenitor at the limbus region [19].The corneal endothelium is not regenerable, corneal dystrophies are therefore mostly due to the endothelial defects or damages [20]. Corneal transplantation is the sole solution for severe corneal wound that lead to LESC diminishing and unreversible corneal dystrophies. In addition, the cornea is an avascular tissue to benefit its transparency. The nearest blood vessels to the cornea are also located in the limbus where an angiogenesis can be initiated by severe corneal damages leading to a pathogenesis called corneal neovascularization. As ZEB1 is required for cell proliferation in the alkali-induced mouse corneal neovascularization model, we show that Zeb1 is highly expressed in corneal epithelial basal cells, vascular endothelial cells, and infiltrated immune cells (Figure 2) [21].

## 3. ZEB1 and Corneal Epithelium

Although the presence of ZEB1 in corneal epithelial cells is evident [22], studies suggest that another zinc finger protein basonuclin (BNC), similar to ZEB1, is a key regulator of the proliferation potential (stemness) of keratinocytes [22]. Tiwari et al. performed a spatiotemporal expression profile of Klf4 in Klf4^∆/∆CE^ corneal epithelium-specific ablation mice. They found that Klf4^∆/∆CE^ cells migrated faster than wild-type cells and showed abnormal stratification, so unable to restore epithelial characteristics. Zeb1 and Zeb2 were up-regulated in Klf4^∆/∆CE^ cornea, which may be related to the role of Klf4 in inhibiting EMT in corneal epithelial homeostasis [23].

Li et al. discovered that SPRR1B was up-regulated by the pro-inflammatory cytokines IL-1 and IFN through the p38 MAPK-mediated signaling pathway, which lead to the activation of the transcription factors CREB and ZEB1, respectively [16]. The SPRR1B induction of p38 MAPK pathway by IFN depends on the ZEB1 response element on the SPRR1B promoter. They proposed a p38-dependent mechanism in which IFN promotes squamous metaplasia through ZEB1-mediated up-regulation of the keratinizing envelope protein SPRR1B. These results revealed that key intracellular signal transduction intermediates participate in the immune-mediated process of ocular surface squamous epithelial metaplasia. This study plows a new avenue for further understanding the significance of ZEB1 in diseases involving corneal differentiation and inflammation, and for potential targeted therapies to prevent abnormal differentiation during chronic corneal inflammation [16].

Ortiz-Melo et al. utilized the rabbit corneal epithelial cell line RCE1 (5T5) as a 3D-culture model, three stages of differentiation were determined according to the growth state of the cultured cells, namely proliferative non-differentiated cells, committed cells, and cells that constitute a stratified epithelium with a limbal epithelial-like structure. They found that the proliferative non-differentiated cells shared characteristics with corneal epithelial stem cells. During this stage, these stem-like cells showed an Oct4+, Klf4+, Myc+, ΔNp63α+, Abcg2+, Vimentin+, Zeb1+, Vangl1+, Krt3-, Krt12- phenotype. They further indicated that Zeb1+ may be related to the difference between corneal epithelium and epidermis and underlying the regulatory mechanism of corneal epithelial cell differentiation [24].

JI-AE is expressed in human corneal epithelial cells, its knockout (KO) increases the expression of ZEB1 in corneal fibroblasts, and this effect in the epithelial cells is mediated by IGF1. Knockdown (KD) of IGF1 by siRNA in the cells further proves that IGF1 secreted by the corneal epithelial cells induces the expression of N-Cadherin in cultured corneal fibroblasts, which is regulated by ZEB1 [25].

## 4. ZEB1 and Corneal Endothelium

ZEB1 plays a key role in EMT by directly regulating several genes related to EMT [26]. However, the function of ZEB1 in corneal endothelium is unknown. Since ZEB1 mutations are identified in endothelial dystrophies, ZEB1 may be involved in retaining endothelial cell density and thereby corneal transparency [27].

### 4.1. Posterior Polymorphous Corneal Dystrophy (PPCD)

PPCD is an autosomal dominant inherited disorder of the corneal endothelium, which is characterized by asymmetric progressive corneal edema and decreased vision. There are three types of PPCD. PPCD type 2 is linked to the mutations in COL8A2, and the underlying genetic disturbance in PPCD type 1 is unknown. PPCD type 3 accounts for about 30% of individuals, caused by a monoallelic mutation of the ZEB1 gene, showing ZEB1 insufficiency in the corneal endothelium [28].

It has been shown that Zeb1 heterozygous and null mouse embryos both show the characteristics of PPCD so that the heterozygous mice can therefore be used as an animal model for PPCD [29]. It is also found that Zeb1 null mouse embryos in late gestation show ectopic expression of epithelial genes in the corneal endothelial and stromal cells, including the basement membrane component Col4a3, similar to the endothelial COL4A3 in PPCD patients [29]. These mouse embryos also showed abnormalities in corneal endothelial and stromal cell proliferation, corneal thickening, and corneolenticular and iridocorneal adhesions [29]. Adult Zeb1 heterozygous mice exhibited less severity of the same defects [29]. The abnormal expression of epithelial genes extends to fibroblasts isolated from Zeb1 heterozygous and null embryos, suggesting that Zeb1 may have a more general role in repression of epithelial properties [29].

To date, a number of nonsense coding region mutations or deletions have been found in the *ZEB1* gene of the PPCD patients [30,31,32,33]. Other data suggest that PPCD3 is caused by ZEB1 haploinsufficiency [28], as a consequence of either the loss of key motifs of nonfunctional ZEB1 protein or dysfunction in the regulation of the mutated ZEB1 activity. ZEB1 truncating mutations result in a significant decrease and/or impaired nuclear localization of the protein, suggesting that ZEB1 sufficiency in PPCD3 may result from protein reduction and/or impaired localization [34]. The ZEB1 mutation led to the loss function of ZEB1-dependent suppression of the epithelial *CDH1* gene. And *COL4A3* expression is repressed by ZEB1 binding to E2 box in the *COL4A3* promoter. The altered expression of *COL4* genes, especially COL4A3, in the corneal endothelium of PPCD3 patients is likely due to the reduced expression of ZEB1 in the setting of a single allele [35].

Frausto et al. reported that a short-interfering RNA (siRNA) targeting ZEB1 to reduce ZEB1 expression in a cell-based model of PPCD lead to increased cell death [36,37], enhanced cell barrier function [38,39,40], as well as corneal endothelial cell apoptosis [41]. These results suggest that the corneal endothelium of PPCD patients not only has abnormal functions, but also has epithelioid phenotype characteristics. Furthermore, they used transcriptomics methods to validate the ZEB1 monoallelic knockout cell line as a cell-based model of PPCD, a new mesenchymal to epithelial transition (MET)-like process called endothelial-to-epithelial transformation (EnET), and proposed the inference of the MET paradigm that explains the PPCD phenotype and the pathogenesis of PPCD [42].

Yellore et al. determined how nonsense mutations in ZEB1 lead to the development of PPCD3. The results showed that both COL4A3 and ZEB1 were expressed in normal human corneal endothelial cells, although in PPCD3, the expression of ZEB1 was reduced compared to the levels of the gene in the healthy control cornea where the expression of COL4A3 was increased, indicating that ZEB1-mediated changes in COL4A3 expression are most likely related to the pathogenesis of the corneal endothelial dystrophy [43].

Aldave et al. demonstrated the expression of ZEB1 in the corneal endothelium and in the corneal stroma. They also showed that the expression of ZEB1 was reduced while the expression of type IV, α3 collagen was increased (COL4A3) in the PPCD3 corneal endothelium, leading to the proposed pathogenesis in affected individuals [44] that resembles the endothelial cell abnormalities observed in mouse *Zeb1* null embryos and heterozygous adult mice [29]. They also found that the proportion of the abnormally steep corneal curvatures was higher in the individuals with PPCD secondary to the ZEB1 deletion. As ZEB1 presents in the nucleus of corneal cells, suggesting ZEB1 may play a role in the development and function of the corneal stroma and endothelium [44].

Two cases of new pleomorphic corneal dystrophy caused by de novo mutation of ZEB1 have been reported, showing congenital corneal endothelial edema [45]. Also, a recent paper finds that missense substitutions in the ZEB1 protein are associated with Fuchs endothelial corneal dystrophy (FECD) and keratoconus, whereas truncated ZEB1 mutations result in PPCD [27]. There is a report that observed the association of triple corneal dystrophy consisting of keratoconus, epithelial basement membrane corneal dystrophy (EBMCD) and FECD [46]. Genetic analysis showed that the TGFb1 mutation was negative, while the heterozygous mutation in the exon 7 of ZEB1 was positive. This is the first case reported in the literature, in which keratoconus, EBMCD and FECD are present in the same patient and are related to ZEB1 gene mutations [46]. They found this situation where all three pathologies existed in the same patient and were related to a mutation in ZEB1. In this study, RT-qPCR was also performed on cultured corneal cells with missense ZEB1 mutation (Gln640His), and identified that COL4A1 and COL4A2 were significantly down-regulated, while COL4A3, COL4A4 and COL8A2 were moderately down-regulated [46].

### 4.2. Fuchs Endothelial Corneal Dystrophy (FECD)

FECD affects about 4% of the population in the United States [47]. It is the most common late-onset, vision-threatening corneal dystrophy. FECD is an age-related disease, mainly in individuals over 40 years of age, especially women. Although the main cause of the disease is not yet known, clinical samples show significant loss of endothelial cells, corneal edema, and thickening of the endothelial basal Descemet’s membrane. It usually follows an inherited autosomal dominant pattern, and its symptoms include decreased vision, hazy cornea, poor night vision, and pain when blinking.

Pan et al. indicated that the high degree of miR-199b-5p methylation may be related to the pathogenesis of FECD. They confirmed that miR-199b-5p directly targets the 3′-UTR of SNAI1 and ZEB1 transcripts and represses their expression and link the downregulation of miR-199b-5p by its methylation to the upregulation of both SNAI1 and ZEB1 [48]. Riazuddin et al. reported a pathogenic mutation of ZEB1 in FECD, but other studies have not shown a significant correlation between ZEB1 and the disease [49]. Gupta et al. conducted a screening of ZEB1 mutations and TCF4 single nucleotide polymorphisms (SNPs) in patients with newly diagnosed FECD in India. They found some novel variants and polymorphisms of ZEB1, and identified TCF4 as a responsible factor for decrease of endothelial cell density [50].

## 5. ZEB1 and Keratoconus

Keratoconus is a progressive thinning of the cornea without inflammation, which can cause severe visual loss due to irregular curvature and scar formation. It can happen alone, but often with other systemic and/or eye diseases. Keratoconus is a recognized genetic disorder. However, the cause of keratoconus is complex, both genetic and environmental factors are at play. The genetic risk factors have been researched genome-wide, among which the corneal dystrophy genes *ZEB1* and *TGFb1* are considered to be related to keratoconus [51].

PPCD and keratoconus have been associated on the same patient cornea in many cases. These reports on ZEB1 gene changes in patients with keratoconus indicate that ZEB1 defects may cause different corneal diseases [51,52]. Other genes like VSX1, SOD1, TGFb1, MIR184, COL4A3/4, RAB3GAP1, LOX, HGF and DOCK9, are also identified in association with keratoconus [51,53].

## 6. ZEB1 and Corneal Wound Healing

Cell proliferation and fibrosis play important roles in a wide variety of physiological processes such as wound healing. In corneal endothelial wound healing, severely affected endothelial cells undergo an endothelial to mesenchymal transition (EnMT), lose some endothelial properties and gain some mesenchymal abnormalities. These cells exhibit proliferative, mobile, and fibrotic properties, which can lead to a reduction in the known reflectivity of the endothelium—loss of corneal transparency.

Lee et al. demonstrated that FGF2 induced the expression of SNAI1, which further activated the expression of ZEB1 and CDK2/cyclin E1 [54]. ZEB1 thereafter induced the mesenchymal phenotype through vimentin, fibronectin, and type I collagen expression. They summarized that FGF2 initiates the EnMT through SNAI1 activation of ZEB1 and CDK2, thereby inducing the production of the matrix proteins COL1A1 and COL1A2 [54]. Lee et al. observed such an EnMT in the mouse corneal endothelium after surgical injury in vivo [54]. They found that a surgical injury induced the expression of Fgf2 and a group of EnMT-related genes like *Snai1, Zeb1, Col1a1, Col1a2, Fn1, Vim, Cdk2* and *Ccne1* in the mouse corneal endothelium. Knockdown of Fgf2 in mouse corneal endothelial cells by siRNA inhibited the injury-dependent expression of Fgf2, Snai1, Zeb1, and Cdh1, indicating that corneal EnMT is under the control of the Fgf2-activated EMT factor Zeb1 [55]. Transient FGF2 stimulation could increase the expression of SP1 and SP3 in human corneal endothelial cells whereas knockdown of ZEB1 by siRNA only reduced the expression of the FGF2-induced SP1 mRNA and protein, but not SP3 [56]. The expression of the FGF2-induced EnMT genes such as *FN1, VIM* and *COLI* was reduced by siRNA knockdown of SP1 and SP3. Compared with SP3, inhibition of SP1 had a greater inhibitory effect. Although SP1 and SP3 proteins were found to interact each other, SP1 and SP3 could bind to the same promoter binding site of EnMT-related genes individually. In addition, siRNA knockdown of Zeb1 inhibited the formation of mouse corneal endothelial injury-dependent fibrosis in vivo. They concluded that ZEB1, through activation of SP1, plays an important role in corneal endothelial fibrosis induced by mesenchymal transition [56].

In the early stage of corneal wound healing, a short process is required to restore the homeostasis of the epidermis, which is reminiscent of EMT. In skin wound healing, this process activates and mobilizes local keratinocytes in the skin to move to the wound bed, thereby re-epithelizing it [57]. ZEB1 is a transcriptional repressor of the epithelial gene E-cadherin [15]. Loss of E-cadherin is known to cause EMT [58,59]. However, in the repair of the corneal epithelium, whether the role of ZEB1 is similar to its role in skin wound repair has not yet been studied.

## 7. ZEB1 and Corneal Neovascularization (NV)

Our previous studies have shown that Zeb1 promotes the development of alkali-induced corneal NV in mice. We have demonstrated that Zeb1 promotes corneal NV by repression of cyclin-dependent kinas (Cdk) inhibitors to promote vascular endothelial cell proliferation; and the loss-of-function of Zeb1, either by the knockdown of mRNA, and by the inhibition of its interaction with Ctbp would inhibit the corneal NV. We also found that the disruption of Zeb1 interaction with Ctbp by the ZEB1–CtBP inhibitors did upregulate the miR-200 family members, but did not repress Vegf expression, indicating that Zeb1 promotes angiogenesis is not through Vegf signal pathway [21].

## 8. ZEB1 and Corneal Inflammation

Less is known regarding the role of ZEB1 in onset and progress of inflammation in the eye. Li et al. discovered that the transcription factors ZEB1 and CREB bind the promoter elements of pro-inflammatory cytokines *IL-1b* and *IFN-γ* and upregulate their expression in corneal epithelial cells through the p38 MAPK-mediated signaling pathway [16]. These results identified ZEB1 and CREB as the key intracellular signaling intermediates involved in the immune-mediated process of ocular surface squamous epithelial metaplasia [16].

Park et al. reported that Epstein-Barr virus (EBV) infection activated Snail and Zeb1, and resulted in their nuclear translocation, potentially leading to diminished epithelial features, enhanced mesenchymal features, and initiation of EMT [60].

## 9. ZEB1 Is a Potential Therapeutical Target

It is extremely hard to treat the corneal genetic disorders that involve a ZEB1 mutation like PPCD. It is theoretically possible to design a gene therapy to locally deliver ZEB1 overexpression vector like adenovirus to the corneal endothelium for a long-term correction of ZEB1 inefficiency. However, no such a trial has so far been reported even in an animal model of PPCD. There may be two reasons: PPCD is a rare ocular disease and it is difficult to control both local infection and expression levels of ZEB1, which may result in unpredictable side effect—possible reactivation of cell division leading to tissue hyperplasia.

Therapeutical methods to reduce ZEB1 expression are also limited because ZEB1 is a transcription factor whose activities usually can not be subdued by small inhibitors. As a result, no direct ZEB1 inhibitor has ever been identified though some indirect small molecules were suggested to inactivate ZEB1 by inhibition of ZEB1 and CtBP interaction such as NSC95397 [61,62]. We have demonstrated that CtBP interaction with ZEB1 retains ZEB1 in the nucleus while disrupt the ZEB1-CtBP complex would transport ZEB1 to the cytoplasm [21]. We have also shown that knockdown of ZEB1 by ZEB1 short hairpin (sh) RNA lentivirus in mouse retinal microvascular endothelial cells significantly reduces their proliferation, and that topical application of the ZEB1-CtBP inhibitor to the alkali-treated mouse corneas decreases corneal neovascularization [21]. These results suggest that both interfering RNA and small chemicals can be of therapeutic significance to reduce expression of ZEB1 in the target corneal cells.

## 10. Conclusions and Future Directions

**Figure 3:**
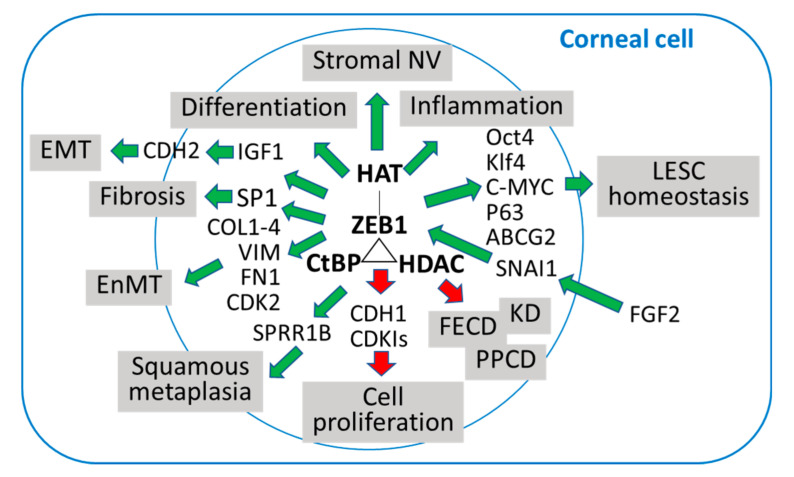
A schematic diagram demonstrating how the transcription factor ZEB1 up- (green arrows) and down- (red arrows) regulates expression of genes and related corneal disorders. HAT, histone acetyltransferase; CtBP, C-terminal binding protein; HDAC, Histone deacetylase; NV, neovascularization; EMT, epithelial to mesenchymal transition; EnMT, endothelial to mesenchymal transition; LESC, limbal epithelial stem cell; KD, Keratoconus; FECD, Fuchs endothelial corneal dystrophy; PPCD, posterior polymorphous corneal dystrophy.

In summary (Figure 3), ZEB1 is an important transcription factor in development; genetic knockout of Zeb1 in mice causes defects in neural, immune and respiratory systems, thereby resulting in embryo death prior to birth [63]. In adults, ZEB1 plays an important role in stem cell homeostasis, immune response, and angiogenesis [21,64,65]. However, abnormal activation may cause cellular transformation like EMT leading to tumorigenesis and fibrosis [8,56]. Therefore, targeting ZEB1 is a potential avenue to generate therapeutics for treatments of various ZEB1-related disorders. Here, we have summarized the presence of ZEB1 and its inefficiency in the cornea that causes corneal endothelial dystrophies, particularly PPCD and FECD. There are many unknowns on ZEB1 in the cornea, particularly the underlying molecular mechanisms for ZEB1 regulation of corneal cell activities during a particular pathogenesis. Our future efforts will continue to dissect the pathways where ZEB1 plays a regulatory role in corneal stem cell homeostasis, immune response, scar formation, and neovascularization.

## Data Availability

Not applicable.
